# Advancing Digital Education Technologies by Empowering Nurses With Point-of-Care Ultrasound: Protocol for a Mixed Methods Study

**DOI:** 10.2196/58030

**Published:** 2024-10-23

**Authors:** Fernanda Raphael Escobar Gimenes, Angelita Maria Stabile, Rodrigo Magri Bernardes, Vinicius Batista Santos, Mayra Gonçalves Menegueti, Patricia Rezende do Prado, Mauricio Serra Ribeiro, Flavia Giron Camerini, Soraia Assad Nasbine Rabeh

**Affiliations:** 1 Ribeirão Preto College of Nursing, University of São Paulo Ribeirão Preto Brazil; 2 Universidade Federal de São Paulo São Paulo Brazil; 3 Faculdade de Medicina de Ribeirão Preto da Universidade de São Paulo Ribeirão Preto Brazil; 4 Universidade do Estado do Rio de Janeiro Rio de Janeiro Brazil

**Keywords:** ultrasound, bedside ultrasound, patient safety, advanced practice nursing, digital technology in education, empowerment, nurses, Point-of-Care Ultrasound, PoCUS, quality care, decision-making, nursing assessment

## Abstract

**Background:**

Bedside ultrasonography, also known as point-of-care ultrasound (PoCUS), is a promising technological tool that enhances clinical assessment, enriching diagnostic capabilities and clinical reasoning. Its use in nursing spans various patient populations and health care settings, providing nurses with a valuable health assessment tool to improve care quality and patient safety. Despite its growing integration into clinical practice, PoCUS training has mainly focused on physicians, leaving a gap for trained nurses who demonstrate similar proficiency in conducting scans and interpreting images. Previous research highlights the value of digital tools in PoCUS training, showing their role in improving professionals’ and students’ knowledge, image interpretation skills, and clinical acumen.

**Objective:**

This study aimed to (1) establish an assessment instrument gauging nurses’ competency milestones in PoCUS and evaluate its content and appearance validity, (2) develop a series of 5 educational videos focused on PoCUS and assess their content and appearance validity, and (3) construct an online learning environment tailored to nurses’ PoCUS training needs and evaluate its content and appearance validity.

**Methods:**

We will conduct a methodological study of technological production guided by Rogers’ diffusion of innovations theory. Subproject 1 will design and validate a comprehensive assessment tool for evaluating nurses’ competency milestones in PoCUS use. For this purpose, a scoping review will be conducted. The review will be based on JBI Collaboration guidelines and reported according to the Preferred Reporting Items for Systematic Reviews and Meta-Analyses extended for Scoping Reviews (PRISMA-ScR) checklist. Subproject 2 involves an evaluation of content and appearance validity for a series of 5 educational videos on PoCUS, designed specifically for nurses about applying peripherally inserted central catheter lines, inserting nasogastric feeding tubes, assessing gastric residual volume, assessing pressure injuries and soft tissue conditions, and assessing muscle mass to monitor patient nutritional status. In subproject 3, a comprehensive online learning environment dedicated to PoCUS training for nurses will be developed and validated. The launch of an online learning environment represents a cornerstone of our dissemination strategy, scheduled to coincide with the inaugural Brazilian PoCUS symposium for nurses, an event organized by the project members. This platform will serve as a pivotal resource for continuous learning and professional development.

**Results:**

Subproject 1 will start in the second half of 2024 and is expected to be completed by mid-2025. Subproject 2 is currently ongoing and is expected to be completed in early 2026. Subproject 3 is set to begin in early 2025 and is planned to be completed by 2026.

**Conclusions:**

Through these concerted efforts, the project aims to bridge the existing gap in PoCUS training for nurses, thereby fostering their proficiency and enhancing patient care outcomes.

**International Registered Report Identifier (IRRID):**

PRR1-10.2196/58030

## Introduction

### Background

Mitigating harm in health care settings is a fundamental duty and responsibility of nurses. The nursing process, with its structured stages, serves as a critical tool in minimizing patient risk by enabling nurses to systematically prioritize health needs and devise outcome-focused care plans [1]. Furthermore, the nursing process embodies the clinical methodology of the nursing profession, enhancing decision-making capabilities and fostering effective communication within the multidisciplinary health care team [2].

The initial phase of the nursing process involves a thorough and accurate data collection or nursing assessment [3,4]. This foundational step is crucial for establishing a comprehensive understanding of the patient’s current health status, which in turn, guides the identification of nursing diagnoses and the determination of outcomes that are responsive to nursing interventions. In this context, nurses leverage technological advancements to bolster their assessments and decision-making processes.

Among these technologies, point-of-care ultrasound (PoCUS) stands out as a particularly promising tool. PoCUS enhances clinical assessments by providing immediate, real-time imaging support for clinical reasoning and diagnosis [5]. This cost-effective and reliable modality generates internal body images using sound waves, with the procedure involving the application of a transducer on the skin’s surface. PoCUS has gained widespread adoption for bedside diagnostics and treatment guidance, offering rapid and efficient solutions [6,7].

Defined as both a portable bedside tool for immediate diagnostic or therapeutic use and a means for the instantaneous acquisition, interpretation, and clinical integration of ultrasound images by health care professionals, PoCUS has transcended its traditional medical confines. Research indicates its use and competency extend to nonmedical professionals [8-13], broadening its application across various clinical settings and care phases, from diagnostic screening to procedure guidance and monitoring [14-16].

The integration of PoCUS into clinical practice has been transformative, equating its diagnostic accuracy with that of conventional imaging methods like x-rays and computerized tomography scans. Its routine application not only reduces the reliance on more invasive tests and patient radiation exposure but also contributes to cost-effective patient care [17,18]. This has led to the proposition of ultrasound as the “fifth pillar” of physical examination, augmenting the traditional quartet of inspection, palpation, percussion, and auscultation [19].

In nursing, the application of PoCUS is diverse, spanning across patient populations [7] and health care levels, from primary [20] to critical [5,11] and emergency care [5,20]. Its use in bedside nursing facilitates the early detection of physiological dysfunctions, thereby enabling more timely and informed clinical decisions crucial for lifesaving interventions [16]. Furthermore, PoCUS complements the physical examination [7,9,13], aids clinical reasoning [5], supports nursing diagnoses, monitors sensitive outcome indicators, and guides invasive procedures [21,22].

In Brazil, the Federal Nursing Council has formally recognized the role of nurses in using bedside ultrasound through Technical Opinion 0052/2021/CTLN/DGEP/COFEn, with specific stipulations regarding its use and the requirement for specialized training. This regulatory endorsement underscores the evolving scope of nursing practice and the integration of advanced technologies like PoCUS in enhancing patient care and outcomes [4].

Despite the increasing recognition of PoCUS as an invaluable tool within nursing practices, a notable gap persists in the literature concerning specialized PoCUS training programs tailored specifically for nurses. While the deployment of PoCUS across various levels of health care underscores its versatility and critical role in enhancing patient care, the existing body of research predominantly focuses on its clinical applications and diagnostic capabilities, rather than on educational frameworks or curricula designed to equip nurses with the requisite skills and knowledge. This discrepancy highlights an educational void, where detailed, nurse-specific training methodologies; competency standards; and assessment criteria remain underexplored. Therefore, this lack of dedicated research into structured PoCUS training programs for nurses not only impedes the standardization of PoCUS proficiency among nurses but also limits the potential for its full integration into nursing care practices. Addressing this gap is essential for ensuring that nurses are adequately prepared to use PoCUS effectively, thereby maximizing its benefits for patient assessment, diagnosis, and management within the nursing scope of practice.

### Primary Objectives

The primary objectives of this study were to (1) design and validate a comprehensive assessment instrument for evaluating nurses’ competency milestones in PoCUS use, (2) develop and disseminate a series of structured educational videos tailored for nurses on the application of PoCUS, and (3) establish a comprehensive online learning environment dedicated to PoCUS training for nurses.

### Secondary Objectives

This study also aimed to (1) conduct a comprehensive analysis of content and appearance validity for the PoCUS competency assessment tool tailored for nurses; (2) undertake a rigorous evaluation of content and appearance validity for a series of 5 educational videos on PoCUS designed specifically for nurses about applying peripherally inserted central catheter (PICC) lines, inserting nasogastric feeding tubes, assessing gastric residual volume, assessing pressure injuries and soft tissue conditions, and assessing muscle mass to monitor patient nutritional status; and (3) assess the content and appearance validity of the online learning environment dedicated to PoCUS training for nurses.

## Methods

### Subproject 1

To achieve the first general and specific objectives, subproject 1 was proposed.

#### Objective

The main objective of this subproject is to establish an assessment tool gauging nurses’ competency milestones in PoCUS and evaluate its content and appearance validity.

This endeavor will undertake a thorough investigation into both the content and appearance validity of the proposed tool, ensuring that it precisely captures the competencies it purports to measure. By adopting a systematic methodology, the study aspires to forge a robust and credible framework for the evaluation of nurses’ proficiency in using PoCUS, making a substantial contribution to the discipline. This endeavor is predicated on the premise that the assessment tool accurately mirrors the quintessential abilities and knowledge indispensable for efficacious clinical application.

In the context of this study, competence is delineated as the consistent and adept execution of a task, seamlessly integrating it into everyday clinical practice. Drawing on the scholarly work of Kumar et al [23], the competency domains identified for effective PoCUS use to encompass a spectrum include (1) the acquisition of foundational knowledge, (2) the practical application of this knowledge, (3) the demonstration of technical and procedural adeptness, (4) the seamless integration of these skills into clinical routines, and (5) the process of certification and recertification to ensure ongoing proficiency and adherence to evolving standards. This comprehensive approach underscores the multifaceted nature of competence in PoCUS, highlighting the importance of a well-rounded and continuous learning process. Through this study, we aim to establish an evaluative mechanism that not only assesses but also promotes the growth of these critical competencies among nurses, thereby enhancing the quality of patient care through the proficient use of PoCUS.

#### Study Design

To curate items for each domain of the proposed assessment tool, a comprehensive scoping review will be orchestrated. The scoping review will be conducted following the JBI Collaboration guidelines and reported in accordance with the Preferred Reporting Items for Systematic Reviews and Meta-Analyses extended for Scoping Reviews (PRISMA-ScR) checklist [24,25].

#### Data Source

This review will leverage a wide array of databases to ensure a thorough exploration of the subject matter. These databases include but are not limited to Cochrane, CINAHL, Embase, PubMed, PsycINFO, Scopus, ERIC, and Scielo. In addition to these repositories, an extensive search of the gray literature will be conducted, encompassing resources such as Google Scholar, as well as repositories of theses and dissertations, to capture a broad spectrum of insights and evidence.

The scoping review will be characterized by its inclusivity, imposing no restrictions on the time frame or language of the studies considered. The inclusion criteria are designed to embrace primary research studies, including review articles, randomized controlled trials, cohort studies, and cross-sectional analyses, while excluding surveys, case reports, textbook chapters, conference proceedings, ongoing research, editorials, and comments. The process of determining relevant descriptors and keywords will be a collaborative effort with a librarian from the University of São Paulo who specializes in systematic reviews, ensuring a comprehensive and targeted search strategy.

Given the objective to acquire a holistic understanding of the field and pinpoint gaps in the existing body of research, the quality of the studies will not be appraised. This process will commence with 2 independent reviewers evaluating studies for inclusion based on their titles and abstracts, adhering to predefined eligibility criteria. In instances where titles and abstracts do not provide sufficient information for preliminary selection, full texts will be examined.

#### Reference Manager

The Rayyan Systems platform will further a double-blind selection process of articles, ensuring an unbiased and efficient review. Discrepancies in the selection process will be reconciled through discussion with a third reviewer.

For organization and analysis, selected articles will be managed using EndNote (version X9, Clarivate Analytics), which will assist in the removal of duplicates and promote the analytical process [26,27].

#### Data Extraction

The data extraction will encompass a diverse range of information, including but not limited to, the name of the instrument, authorship details, the country of development, language, publication year, constructs measured, target population, mode of instrument application, scale content and item number, response formats, and scoring methods (Multimedia Appendix 1). Furthermore, a detailed examination of the psychometric properties of each instrument will be conducted, with findings presented in tabular format. Additional data pertaining to the objectives, design, interventions, participants, sample sizes, and outcomes of the studies will also be extracted and systematically organized in tables, enhancing the clarity and accessibility of the evidence synthesized [28].

The review will be registered with PROSPERO (International Prospective Register of Systematic Reviews) in the second semester of 2024.

Drawing upon the comprehensive insights garnered from the scoping review, a designed assessment tool will be developed to evaluate nurses’ competency milestones throughout their PoCUS training journey. This tool will be intricately aligned with the competency domains delineated by Kumar et al [23], ensuring a robust framework that encapsulates essential skills and knowledge. Following its development, the instrument will be subjected to a validation process, involving an evaluation by a professional proficient in Portuguese and a panel of nurses who boast significant experience in PoCUS-related care, education, and research. The Delphi technique [29], renowned for its structured communication process and consensus-building capabilities, will be used to expedite this evaluation, ensuring a comprehensive and iterative assessment.

Given the nascent stage of PoCUS use among Brazilian nurses and the limited pool of experts in this domain, the selection of experts will leverage the snowball sampling technique [30]. This method is particularly suited to the context, allowing for the identification and recruitment of key informants through the networks of initial participants, thereby ensuring a panel enriched with specialized knowledge and experience. Participants who do not return the completed assessment tools within a stipulated 30-day period will be considered nonresponsive and thus excluded from the study.

The recruitment of experts will be conducted through intentional nonprobabilistic sampling, emphasizing the deliberate selection of individuals based on their expertise and experience in PoCUS. Upon their voluntary agreement to partake in the research, evidenced by written consent, these experts will embark on a critical evaluation of the assessment tool. Their scrutiny will focus on the clarity of indicators, their theoretical relevance, and practical applicability, using a dichotomous scale of “agree” or “disagree” for each item. This systematic evaluation process is designed to ensure that each item on the assessment tool is clear, relevant, and practically applicable to the context of nursing practice.

To ascertain the content validity of the tool, the content validity ratio (CVR) for each indicator will be calculated. The CVR, a statistical measure of content validity, will serve as a quantitative gauge of the extent to which the items are deemed essential by the panel of experts [31]. The acceptance threshold for the CVR will be determined based on the number of experts participating in the assessment, adhering to established methodological standards. This validation process aims to establish an accepted assessment tool that accurately reflects the competencies necessary for effective PoCUS application by nurses, thereby contributing to the enhancement of training and practice in this evolving domain [31,32].

### Subproject 2

To achieve the second general and specific objectives, subproject 2 was proposed.

#### Objective

The main objective of this subproject is to develop a series of 5 educational videos focused on PoCUS and assess their content and appearance validity.

#### Study Design

This methodological investigation uses a cross-sectional design, underpinned by the theoretical perspectives of Fleming et al [33]. It is set to unfold at the Ribeirão Preto College of Nursing, University of São Paulo, an institution renowned for its Nursing Practice Simulation Center, a state-of-the-art recording studio, and a dedicated multimedia team.

Given the emergent status of PoCUS expertise among Brazilian health care professionals, experts will be identified through a snowball sampling technique [30], ensuring a comprehensive representation of knowledge in this nascent field. Participants who fail to return their assessments within a 30-day window will be respectfully excluded from the study, emphasizing the importance of timely feedback. Recruitment will be intentional and nonprobabilistic, targeting individuals who demonstrate a profound understanding and experience in PoCUS. Those agreeing to partake in the study will be required to sign an informed consent form, marking their voluntary commitment to this scholarly endeavor.

The research is structured into distinct phases, each designed to ensure the elaboration and validation of high-quality educational content.

#### Phase I: Preproduction

This initial phase involves a narrative literature review focused on the procedural skills necessary for the ultrasound-guided insertion of PICC lines and nasogastric feeding tubes. In addition, it will explore the use of ultrasound in assessing gastric residual volume, pressure injuries and soft tissue conditions, and muscle mass, particularly in relation to patient nutritional status. This comprehensive review will span several databases including CINAHL, Embase, LILACS, PubMed, and Scopus, without constraints on publication date or language. Furthermore, textbooks and both national and international guidelines will be consulted to ensure a thorough understanding of the subject matter, including content about the physical principles of ultrasound.

#### Phase II: Production

Informed by the insights gathered in phase I, this phase involves the development of scripts and storyboards for the educational videos, adhering to the best practices as recommended by Fleming et al [33]. PoCUS experts, including physicians and nurses, will be invited to contribute to this creative process, ensuring the content’s relevance and accuracy. Following previous studies [34-36], the Delphi technique [29] will be used to refine the scripts and storyboards, with participants providing their expertise through a structured, iterative consultation process.

To evaluate the content validity evidence of the scripts and storyboards for the educational videos, a tool, refined from Ferreira’s [37] methodology, will be used (Multimedia Appendix 2). This comprehensive tool encompasses 6 pivotal categories, namely objectives, content, relevance, environment, verbal language, and inclusion of topics, ensuring a holistic assessment of the educational materials. Each category will be scrutinized using a nuanced 4-point Likert scale, where a rating of 4 signifies “strongly agree,” 3 denotes “agree,” 2 indicates “disagree,” and 1 represents “strongly disagree.” In addition, the tool thoughtfully includes a section for experts to articulate their detailed opinions and constructive suggestions on each evaluated item, fostering a rich, collaborative refinement process.

Upon the completion of this assessment by the panel of experts, a collaborative scheduling process will be initiated with the multimedia department of Ribeirão Preto College of Nursing, University of São Paulo, to plan the filming sessions. These sessions will take place in the Nursing Practice Simulation Center, ensuring a conducive environment for high-quality video production. The filming will necessitate the use of a portable ultrasound device, equipped with both linear and convex transducers, to accurately depict the procedures. In addition, simulators will be used to promote the realistic demonstration of the insertion of PICC lines and nasogastric tubes, guided by ultrasound technology. To further enhance the realism and educational value of the videos, volunteers will be recruited to portray patients, providing authentic scenarios for the nurses. These nurses will demonstrate the invasive procedures using simulators or clinical assessments using volunteers, showcasing practical applications of the skills being taught.

#### Phase III: Postproduction

This final phase focuses on the editing and polishing of the educational videos. The Ribeirão Preto College of Nursing, University of São Paulo, multimedia department will lend their expertise in video editing, using advanced software to enhance the educational content with graphic animations, text, images, and sound. Professional voice-over narration will be recorded to accompany the visuals, ensuring clarity and engagement. Upon completion, the videos will undergo a validation process, assessing both content and appearance validity with the help of PoCUS experts and audiovisual technicians.

Throughout this process, the project will leverage the Moodle system of the University of São Paulo [38] to enhance communication and collaboration among the experts. This online learning environment will host the scripts, storyboards, and validation tools, providing a centralized platform for the validation process. To evaluate the appearance of the educational materials, the study will implement the Instrument for Appearance Validity of Educational Technology in Health (IVATES) [39] (Multimedia Appendix 3). This tool comprises 12 targeted questions, each measured against a detailed 5-point Likert scale for nuanced feedback. The scale ranges from 1, indicating “completely disagree,” to 5, representing “completely agree,” allowing experts to precisely express their assessment of the educational content’s effectiveness and aesthetic appeal.

To tailor this tool specifically for the unique demands of this study, thoughtful adaptations will be made. Notably, the original term “illustrations” will be substituted with “video segments” to better reflect the multimedia nature of the content being evaluated. Similarly, “figures” will be replaced with “images” to more accurately describe the visual elements used within the educational videos. In addition, a pivotal modification will be made to question 12, which will now focus on “assistance in teaching-learning procedures.” This change shifts the emphasis from assessing attitudinal changes to evaluating the direct educational impact of the videos, aligning the instrument more closely with the study’s objectives.

#### Data Analysis

Data characterizing PoCUS experts will be stored in the University of São Paulo Moodle platform for subsequent download into Microsoft Excel spreadsheets. They will then be transferred to the SPSS (version 26.0; IBM Corp). Descriptive statistics will be used for analysis, including measures of central tendency and dispersion for quantitative variables, and absolute and relative frequencies for categorical variables. Data normality will be checked using the Shapiro-Wilk test, with a distribution considered normal when *P*>.05.

During the evaluation stage of the video’s content validity, the CVR will be calculated according to Ayre and Scally [31]. The CVR ranges from –1 to +1, with positive values indicating that more than half of the experts deemed the item essential. Ayre and Scally [31] proposed a table with minimum CVR values based on the number of experts, to determine an item’s acceptability at a 5% significance level.

For the evaluation of the appearance of the videos, the appearance validity index (AVI) will be used, where the AVI for each item (AVI-I) will be calculated by dividing the number of experts who rate the item as 4 or 5 by the total number of participants. For the total AVI (AVI-T), the sum of the AVI-I values will be divided by the total number of items. An item with an AVI≥0.78 will be considered excellent; values between 0.60 and 0.77 will indicate the need for adjustments to improve the appearance of the educational health technology; an item with an AVI<0.60 will be classified as unsatisfactory, and the material will need to be revised starting from the key point of the item. The AVI-T should be ≥0.90 [39]. Data analysis will be conducted by a statistician from the Ribeirão Preto College of Nursing, University of São Paulo.

### Subproject 3

#### Objective

The main objective of this subproject is to construct an online learning environment tailored to nurses’ PoCUS training needs and evaluate its content and appearance validity.

#### Study Design

This cutting-edge methodological study in nursing education is grounded in the seminal work of Rogers’ [40] diffusion of innovations theory. At the heart of this theory is the concept of innovation, defined as a novel idea, practice, knowledge, or object that is perceived as new by an individual or community and is integrated into their practices, leading to significant behavioral transformations. For such innovations to gain widespread acceptance and integration, they should be disseminated through effective communication channels that ensure easy accessibility. A prime example of this is the online learning environment, which transcends the traditional constraints of time and space, thereby promoting a seamless and impactful implementation of innovative educational strategies.

The process of adopting an innovation is delineated into five distinct stages [40]: (1) knowledge, the stage at which an individual gains awareness and understanding of the innovation; (2) persuasion, the stage where an individual develops a favorable attitude towards the innovation; (3) decision, the critical juncture at which an individual decides whether to embrace or reject the innovation; (4) implementation, the phase where the individual actively starts using the innovation, signifying a behavioral shift; and (5) confirmation, the final stage where the individual reaffirms their commitment to the innovation and fully integrates it into their practice. This study will delve into the initial 3 stages to uncover the dynamics of innovation adoption within the context of nursing education.

To further the development of the online learning environment, the instructional design will adhere to the comprehensive “ADDIE” model, which encompasses 5 pivotal phases: analyze, design, develop, implement, and evaluate [41].

#### Analysis Phase

Recognizing the nascent yet growing importance of ultrasound use in nursing, this phase will be addressed through subprojects 1 and 2, laying the groundwork for an informed instructional strategy.

#### Design Phase

The online learning environment will be crafted with learning objectives that span a broad spectrum of ultrasound applications in nursing. These objectives will include (1) mastering ultrasound-guided PICC line insertion, (2) learning ultrasound-guided nasogastric tube insertion techniques, (3) conducting qualitative and quantitative assessments of gastric residual volume to avert bronchopulmonary aspiration through PoCUS, (4) evaluating pressure injuries and soft tissue conditions using PoCUS, and (5) assessing muscle mass to monitor patient nutritional status with a focus on PoCUS.

#### Development Phase

The online learning environment will be structured into 5 comprehensive modules, each designed to address the learning objectives outlined. These modules will incorporate instructional videos that elucidate basic ultrasound principles and demonstrate procedural techniques essential for effective examinations, developed as part of subproject 2. To enrich the learning experience, the modules will also include recommended additional readings, anatomical landmark flashcards for quick recognition, educational quizzes to reinforce key concepts, pre- and post–self-assessment tests to gauge learning progress, and clinical case studies. These case studies are designed to foster clinical reasoning skills, with a strong emphasis on the nursing process and the integration of standardized nursing language systems, including NANDA International, Inc [42], Nursing Outcomes Classification (NOC) [43], and Nursing Interventions Classification (NIC) [44]. The modules will be created by experts in PoCUS and nursing education.

#### Implementation

During the implementation phase, the online learning environment will be evaluated through both content and appearance validity evidence analysis, ensuring its educational efficacy and user engagement. The content validity will be assessed by a panel of physicians and nurses, each boasting expertise in the relevant subject matter. This evaluation will be expedited through the use of the content validity index (CVI) [31,32], with experts being carefully selected through intentional nonprobabilistic sampling methods to ensure a comprehensive and informed review process. Similarly, the appearance validity will be scrutinized by professionals well-versed in the domain, using the IVATES tool [39] (Multimedia Appendix 3) to guarantee a thorough appraisal of the online learning environment’s instructional design and content relevance.

In a strategic move to enhance the evaluation process, this project will incorporate the evaluation tool developed by Ferreira [37] (Multimedia Appendix 2), which categorizes the assessment criteria into 6 distinct domains: objectives, content, relevance, environment, verbal language, and inclusion of topics. Each category will be evaluated using a 4-point Likert scale, ranging from “strongly agree” to “strongly disagree,” providing a nuanced framework for feedback. In addition, this tool thoughtfully allows experts to articulate their reasoning for any disagreements, further enriching the feedback with detailed insights and constructive suggestions for each evaluated item.

#### Evaluation Phase

Upon the completion of the content and appearance validity evidence analysis by the panel of experts, the online learning environment will transition to a pilot testing phase, engaging a select group of nurses to interact with and evaluate the system. Their experiences and perceptions will be captured through the System Usability Scale, Brazilian version [45] (Multimedia Appendix 4), an instrument comprising 10 items, scored on a scale from “strongly disagree” to “strongly agree.” This scale not only aligns semantically, idiomatically, conceptually, and culturally with its original English counterpart but also demonstrates exceptional reliability and structural construct validity, ensuring a credible and comprehensive assessment of the online learning environment’s usability.

#### Data Analysis

The content validity evidence for the online learning environment will be quantitatively assessed through the calculation of the CVI, with items receiving scores of 4 or 5 by experts deemed positively validated. The criteria for validity will follow established benchmarks, with a CVI≥0.78 indicating excellent validity, scores between 0.60 and 0.77 classified as good, and scores <0.59 considered poor [39,46].

To determine the overall usability score derived from the application of the System Usability Scale, Brazilian version [45], a calculation process will be used. For odd-numbered statements, the score assigned by the evaluator will be reduced by 1, whereas for even-numbered statements, it will be reduced by 5. The aggregated individual scores will then be multiplied by 2.5 to obtain the total scale value. This final score will speed a nuanced interpretation of the online learning environment’s usability, ranging from the worst imaginable scenario to an ideal scenario, thus providing a comprehensive understanding of the user’s perception and the system’s overall performance. Data analysis will be conducted by a statistician from the Ribeirão Preto College of Nursing, University of São Paulo.

### Ethical Considerations

The project was submitted for ethical review to the Research Ethics Committee of the Ribeirão Preto College of Nursing, University of São Paulo, in November 2023 (Institutional Review Board approval 78296623.2.0000.5393). This submission underscores our commitment to conducting the study in strict adherence to Resolution 466, dated December 12, 2012, issued by the National Council of Ethics in Research of the Ministry of Health. In addition, all participants will be furnished with an informed consent form (Multimedia Appendix 5).

It is important to note that participation in this study will not entail any financial burden on the participants, nor will it offer any direct monetary incentives. The anticipated risks associated with participation are deemed minimal, primarily concerning the potential inconvenience due to the time investment required for engaging with the research tools. To alleviate any potential discomfort, we will offer participants the flexibility to complete the tools at their own pace, in multiple sittings if preferred.

In the event of any unforeseen harm arising from participation in the study, we are fully prepared to uphold the participants’ rights to compensation, in alignment with the prevailing legal standards of the country. This assurance is extended by both the research team and the sponsoring institution, highlighting our commitment to participant welfare.

Furthermore, we acknowledge the inherent risks associated with the digital environment, particularly the limitations of current technologies that may impede our ability to ensure absolute confidentiality and the potential risk of data breaches. Participants will, therefore, be advised to retain a copy of all electronic communications for their records. In an effort to mitigate these risks, upon the conclusion of the study, all collected data will be securely transferred to a password-protected computer, accessible solely to the project team members. Subsequently, all data stored on the online platform will be permanently erased.

While the direct involvement of specialists in this study may not yield immediate personal gains, it is anticipated to offer significant indirect and long-term professional benefits. The educational materials developed, including videos and the online learning environment, are expected to substantially enhance nurses’ comprehension of the subject matter, thereby promoting future scholarly endeavors. Furthermore, the potential benefits of this study extend beyond the immediate academic community, promising to effectuate enduring improvements in nursing care for adult patients across all health care settings. This endeavor thus represents a commitment to ethical research conduct, participant protection, and the advancement of nursing education and patient care on a broader scale.

## Results

Subproject 1 will start in the second half of 2024 and is expected to be completed by mid-2025. Subproject 2 is currently ongoing and is expected to be completed in early 2026. Subproject 3 is set to begin in early 2025 and is planned to be completed by 2026.

## Discussion

### Expected Findings

Previous studies have highlighted the importance of PoCUS in clinical settings, particularly in enhancing diagnostic accuracy [12,22,47,48] and patient outcomes [49-52]. However, there has been a lack of standardized tools to assess nurses’ competency in using PoCUS. This study builds on the foundational work of researchers like Fleming et al [33], who have emphasized the need for structured educational materials and competency assessments. By incorporating a scoping review and expert consultations, this research addresses the gaps identified in previous studies and will offer a validated assessment tool [32,33].

The results will be showcased through presentations at undergraduate and master’s thesis defenses, marking a significant contribution to the academic discourse. Furthermore, to ensure the findings are accessible to a diverse audience, they will be published in esteemed journals within the field. A deliberate focus will be placed on journals indexed in prestigious databases such as the ISI Web of Science and Scopus, with publications available in Portuguese, English, and Spanish.

The strengths of this study include its comprehensive methodology, which uses a scoping review following JBI Collaboration guidelines to ensure a thorough exploration of the subject matter. The involvement of PoCUS experts, including physicians and nurses, in the development of scripts and storyboards enhances the content’s relevance and accuracy. In addition, the iterative validation process, which uses the Delphi technique for refining educational materials, ensures that the content is rigorously evaluated and validated by experts. However, there are limitations to consider. The study is conducted within a specific institutional context (Ribeirão Preto College of Nursing, University of São Paulo), which may limit the generalizability of the findings to other settings. The development and validation processes are resource-intensive, requiring significant time and expert involvement. Furthermore, the reliance on expert opinions in the Delphi technique may introduce bias, although this is mitigated through structured, iterative consultations [29].

Subproject 2 focuses on the creation and validation of 5 educational videos specifically designed for nurses. The expected results from this subproject include the development of high-quality, validated educational videos that can be integrated into nursing curricula. These videos aim to enhance nurses’ practical skills and confidence in using PoCUS, ultimately improving patient care. The results will be showcased through presentations at undergraduate and master’s thesis defenses and will be published in esteemed journals. Summarizing key findings and insights will be produced and disseminated through YouTube, complemented by strategic promotions through social media channels such as Facebook (Meta) and Instagram (Meta), as well as on the official websites of the participating universities. Recognizing the importance of linguistic diversity and inclusivity, one of the videos will be specially crafted in Spanish to cater to the Spanish-speaking audience. This initiative will be bolstered by the support from Sigma (Latin America and Caribbean Region), leveraging the unique position of a Chilean group member who is currently a master’s student in the Fundamental Nursing Graduate Program at Ribeirão Preto College of Nursing, University of São Paulo.

Subproject 3 aims to construct and evaluate evidence of content and appearance validity for an online learning environment tailored to nurses’ PoCUS training needs. This potent education resource is designed to foster clinical reasoning skills with a strong emphasis on the nursing process and the integration of standardized nursing language. The evaluation phase will involve pilot testing the online learning environment with a select group of nurses, capturing their experiences and perceptions through the System Usability Scale, Brazilian version. The results from this subproject will provide valuable insights into the usability and effectiveness of the online learning environment in enhancing PoCUS training for nurses.

Future research should focus on assessing the long-term impact of these educational videos and the online learning environment on nursing practice and patient outcomes. In addition, exploring the implementation of these educational resources in diverse health care settings will help evaluate their effectiveness and adaptability. Investigating the integration of advanced technologies, such as online reality and artificial intelligence, to enhance the educational experience is another promising area for future research.

To ensure the findings are accessible to a diverse audience, the results will be disseminated through multiple channels. Academic publications in esteemed journals indexed in prestigious databases such as ISI Web of Science and Scopus will be pursued. The results will also be presented at undergraduate and master’s thesis defenses, as well as at national and international conferences. Workshops and training sessions will be organized for nurses and health care professionals to share the validated assessment tool, educational videos, and online learning environment. In addition, online platforms and social media will be used to reach a broader audience and facilitate knowledge sharing and collaboration. Key findings and insights will be disseminated through YouTube and promoted through social media channels such as Facebook and Instagram, as well as on the official websites of the participating universities.

### Conclusions

The anticipated outcomes of this research underscore its potential to significantly enhance the competency of nurses in using PoCUS through the development and validation of a robust assessment tool. By addressing the current gaps in standardized competency evaluations, this study aims to elevate the quality of nursing education and clinical practice. The creation of high-quality educational videos and a tailored online learning environment is expected to provide nurses with practical, accessible, and engaging resources that foster both skill acquisition and confidence in PoCUS procedures. These educational interventions are poised to improve patient care outcomes by ensuring that nurses are well equipped to perform PoCUS with proficiency.

Furthermore, the dissemination of the findings through various academic and public channels will promote widespread adoption and integration of these resources into nursing curricula globally. The iterative validation process involving PoCUS experts ensures the relevance and accuracy of the educational content, while the use of advanced methodologies like the Delphi technique and structured scoping reviews guarantees a rigorous evaluation framework.

In conclusion, this research protocol not only aims to fill a critical void in PoCUS competency assessment but also aspires to set a new standard in nursing education. By leveraging innovative educational tools and methodologies, the study promises to contribute to the ongoing professional development of nurses and ultimately enhance patient care across diverse health care settings. The commitment to ethical research conduct and participant protection further strengthens the study’s foundation, ensuring that the outcomes are both scientifically sound and socially responsible.

## Appendix

Multimedia Appendix 1 Data extraction tool.

Multimedia Appendix 2 Validation of the educational video script tool.

Multimedia Appendix 3 IVATES (Instrument for Appearance Validity of Educational Technology in Health) tool.

Multimedia Appendix 4 System Usability Scale, Brazilian version.

Multimedia Appendix 5 Informed consent forms.

## Abbreviations

AVI: appearance validity index
AVI-I: appearance validity index for each item
AVI-T: total appearance validity index
CVI: content validity index
CVR: content validity ratio
IVATES: Instrument for Appearance Validity of Educational Technology in Health
NIC: Nursing Interventions Classification
NOC: Nursing Outcomes Classification
PICC: peripherally inserted central catheter
PoCUS: point-of-care ultrasound
PRISMA-ScR: Preferred Reporting Items for Systematic Reviews and Meta-Analyses extended for Scoping Reviews
PROSPERO: International Prospective Register of Systematic Reviews
