# Supplementary material for: Advancing Digital Education Technologies by Empowering Nurses With Point-of-Care Ultrasound: Protocol for a Mixed Methods Study
Source: JMIR Res Protoc. 2024 Oct 23;13:e58030. doi: 10.2196/58030 (PMC11541147; doi:10.2196/58030)
Supplement: Multimedia Appendix 1 [file resprot_v13i1e58030_app1.pdf]

JBI Template for Data Extraction[1]

SCOPING REVIEW DETAILS													
**Scoping Review title:**													
**Review objective/s:**													
**Review question/s:**													
**ID**	**Title**	**Author(s)**	**Year**	**Country**	**Journal**	**Objective(s)**	**Context**	**Method**	**Participants**	**Sample**	**Domains of the instrument**	**N of items**	**Details of the psychometric analysis**

[1] JOANNA BRIGGS INSTITUTE. JBI template source of evidence details, characteristics and results extraction instrument. Available at: https://jbi-global-wiki.refined.site/space/MANUAL/4687579/Appendix+11.1+JBI+template+source+of+evidence+details%2C+characteristics+and+results+extraction+instrument. Access in: 24 oct. 2023.
