# Supplementary material for: Advancing Digital Education Technologies by Empowering Nurses With Point-of-Care Ultrasound: Protocol for a Mixed Methods Study
Source: JMIR Res Protoc. 2024 Oct 23;13:e58030. doi: 10.2196/58030 (PMC11541147; doi:10.2196/58030)
Supplement: Multimedia Appendix 2 [file resprot_v13i1e58030_app2.pdf]

Validation of the Educational Video Script[1]

Instructions:

Dear specialist, we kindly ask you to evaluate the **educational video** (which is available in this virtual learning environment) according to the following aspects:

1. Functionality
2. Usability
3. Efficiency
4. Audiovisual technique
5. Environment
6. Procedure

Next, please answer the questions by marking the column that best represents the degree achieved in each criterion.

1. FUNCIONALITY: refers to the functions and/or objectives of the educational video aimed at facilitating the teaching of Point-of-Care Ultrasound (or POCUS) by nurses in adult patients.

	**Strongly Agree**	**Agree**	**Disagree**	**Strongly Disagree**	**Don't Konw**
1.1 The video presents itself as an appropriate tool for the intended objective.					
1.2 The video enables the generation of positive results in the teaching-learning process on the subject.					

Recommendation:

____

____

[1] FERREIRA, Maria Verônica Ferrareze. Dressing of central venous catheters: supports for nursing teaching and care. 2013. 228 p. Dissertation (Doctoral) - University of São Paulo at Ribeirão Preto College of Nursing, Ribeirão Preto, 2013. doi:10.11606/T.22.2013.tde-26092013-185000. Access in: 2023-10-24

2. USABILITY: It refers to the effort required to use the video, as well as the individual judgment of that use.

	**Strongly Agree**	**Agree**	**Disagree**	**Strongly Disagree**	**Don't Konw**
2.1 The video is easy to use.					
2.2 It is easy to learn the theoretical concepts used and their applications.					
2.3 It allows the user to easily apply the concepts worked on in clinical practice.					

Recommendation:

____

____

3. EFFICIENCY: refers to the performance level of the video and the amount of resources used (related to time) under established conditions.

	**Strongly Agree**	**Agree**	**Disagree**	**Strongly Disagree**	**Don't Konw**
3.1 The duration of the video (time used) is appropriate for the user to learn the content.					
3.2 The number of scenes is consistent with the proposed time for the video.					

Recommendation:

____

____

4. AUDIOVISUAL TECHNIQUE: refers to the set of technical resources employed for the presentation of the video content.

	**Strongly Agree**	**Agree**	**Disagree**	**Strongly Disagree**	**Don't Konw**
4.1 The lighting is adequate for observing the scenes.					
4.2 The narrator's tone and voice are clear and appropriate.					
4.3 The video narration is used efficiently and is understandable to the user.					
4.4 It is possible to return to any part of the scenes when desired.					

Recommendation:

____
____

5. ENVIRONMENT: refers to the evaluation of the location where the educational video was filmed.

	**Strongly Agree**	**Agree**	**Disagree**	**Strongly Disagree**	**Don't Konw**
5.1 The video reflects the daily routine of clinical practice.					
5.2 The laboratory environment did not interfere with the fidelity of the invasive procedure or clinical assessment.					

Recommendation:

____
____

6. PROCEDURE: evaluates whether the content presented in the educational video allows for the understanding of the items listed below.

	**Strongly Agree**	**Agree**	**Disagree**	**Strongly Disagree**	**Don't Konw**
6.1 Objectives of the educational video.					
6.2 Legislation on the use of ultrasound by nurses.					
6.3 Purpose of bedside ultrasound.					
6.4 Objective(s) of the video.					
6.5 A complete presentation of the materials used in the procedure was provided.					
6.6 The steps of the procedure are appropriate and could be identified.					

Recommendation:

____
____
