# Supplementary material for: Advancing Digital Education Technologies by Empowering Nurses With Point-of-Care Ultrasound: Protocol for a Mixed Methods Study
Source: JMIR Res Protoc. 2024 Oct 23;13:e58030. doi: 10.2196/58030 (PMC11541147; doi:10.2196/58030)
Supplement: Multimedia Appendix 3 [file resprot_v13i1e58030_app3.pdf]

INSTRUMENT FOR APPEARANCE VALIDITY OF EDUCATIONAL TECHNOLOGY IN HEALTH (IVATES)[1]

To respond to this instrument, you will need to select one of the options 1, 2, 3, 4, or 5, which have the following meanings:

1 = TOTALLY DISAGREE
2 = DISAGREE
3 = PARTIALLY DISAGREE
4 = AGREE
5 = TOTALLY AGREE.

Items	1	2	3	4	5
	Strongly disagree	Disagree	Partially disagree	Agree	Strongly agree
1. Illustrations are suitable for the target audience.					
2. Illustrations are clear and easy to understand.					
3. Illustrations are relevant for the content understanding by the target audience.					
4. The colors of illustrations are suitable for the type of material.					
5. The shapes of illustrations are suitable for the type of material.					
6. Illustrations depict the daily life of the target audience of the intervention.					
7. The layout of figures is in harmony with the text.					
8. The pictures used elucidate the content of the educational material.					
9. Illustrations help to expose the theme and are in a logical sequence.					
10. Illustrations are in appropriate quantity in the educational material.					
11. Illustrations are in appropriate size in the educational material.					
12. Illustrations help to change the behavior and attitudes of the target audience.					

Observations:

____

____

[1] SOUZA, A. C. C. DE .; MOREIRA, T. M. M.; BORGES, J. W. P. Development of an appearance validity instrument for educational technology in health. Revista Brasileira de Enfermagem, v. 73, p. e20190559, 2020. Available at: https://www.scielo.br/j/reben/a/j4nNFSCVRjLFkTfXYBkLWgk/?format=pdf&lang=en. Access in: 29 sep. 2024.
