# Supplementary material for: Advancing Digital Education Technologies by Empowering Nurses With Point-of-Care Ultrasound: Protocol for a Mixed Methods Study
Source: JMIR Res Protoc. 2024 Oct 23;13:e58030. doi: 10.2196/58030 (PMC11541147; doi:10.2196/58030)
Supplement: Multimedia Appendix 4 [file resprot_v13i1e58030_app4.pdf]

User Experience Questionnaire – Portuguese Version[1]

For each of the statements below, select the box (only one per line) that best describes your reaction to the virtual learning environment you have just accessed.

**Item**	**Strongly disagree**	**Partially disagree**	**Neither disagree nor agree**	**Partially agree**	**Strongly agree**
	**1**	**2**	**3**	**4**	**5**
1 - I think that I would like to use this system frequently.					
2 - I found the system unnecessarily complex.					
3 - I thought the system was easy to use.					
4 - I think that I would need the support of a technical person to be able to use this system.					
5 - I found the various functions in this system were well integrated.					
6 - I thought there was too much inconsistency in this system.					
7 - I would imagine that most people would learn to use this system very quickly.					
8 - I found the system very cumbersome to use.					
9 - I felt very confident using the system.					
10 - I needed to learn a lot of things before I could get going with this system.					

[1] LOURENCO, D.F.; CARMONA, E.V.; LOPES, M.H.B.M. Translation and Cross-Cultural Adaptation of the System Usability Scale to Brazilian Portuguese. Aquichan [online]. 2022, v.22, n.2, e2228. Epub May 13, 2022. Available at: https://doi.org/10.5294/aqui.2022.22.2.8. Access in: 24 oct. 2023.
