# Supplementary material for: Advancing Digital Education Technologies by Empowering Nurses With Point-of-Care Ultrasound: Protocol for a Mixed Methods Study
Source: JMIR Res Protoc. 2024 Oct 23;13:e58030. doi: 10.2196/58030 (PMC11541147; doi:10.2196/58030)
Supplement: Multimedia Appendix 5 [file resprot_v13i1e58030_app5.pdf]

UNIVERSIDADE DE SÃO PAULO
ESCOLA DE ENFERMAGEM DE RIBEIRÃO PRETO

Centro Colaborador da OPAS/OMS para o Desenvolvimento da Pesquisa em Enfermagem

Avenida Bandeirantes, 3900 - Ribeirão Preto - São Paulo - Brasil - CEP 14040-902
Fone: 55 16 3315.3382 - 55 16 3315.3381 - Fax: 55 16 3315.0518
www.eerp.usp.br - eerp@usp.br

Informed Consent Form (ICF) Analysis by Experts of the Nurse Competency Assessment Instrument in PoCUS

You are being invited to participate as an expert in stage 2 of the research titled "Point-of-Care Ultrasound for Nurses: Development and Validation of Digital Educational Technologies." This study is conducted by Prof. Dr. Fernanda Raphael Escobar Gimenes from the School of Nursing of Ribeirão Preto at the University of São Paulo (EERP-USP). The aim is to develop and validate an instrument to assess nurses' competencies in PoCUS (Point-of-Care Ultrasound), also known as bedside ultrasound.

To achieve our goals, we invite you to evaluate the "Nurse Competency Milestones Assessment Instrument in PoCUS." Your feedback on the clarity of the items, theoretical relevance, and practical pertinence will be requested using a dichotomous scale (agree and disagree). You will receive a copy of the instrument by email for analysis and feedback, which should be completed and returned within 30 days of receipt. The estimated time for the evaluation is approximately 40 minutes, at a day and time of your convenience.

The risks of this research are minimal and are mainly related to expressing your opinion and the time spent responding to the instrument's evaluation questionnaire. If you feel uncomfortable expressing your opinion, the researcher will be available to address your discomfort. Additionally, there are inherent risks in the virtual environment, such as technological limitations. However, at the end of the research, the information will be downloaded to a password-protected device accessible only to the researchers. The data on the virtual platform will be deleted. In case of damages resulting from participation in the research, we guarantee the right to compensation according to the laws in force in the country (Item IV.3-h – CNS Resolution 466/2012).

Your participation is voluntary and will not bring immediate personal benefits, but it will contribute to the knowledge in the field of nursing and may positively impact future patient care involving PoCUS procedures.

It is important to note that if at any time you decide not to participate in the research, you can withdraw your consent without any personal prejudice. Additionally, you have the right to request the complete deletion of your information from the study. Furthermore, there will be no costs or remuneration for participation.

The information collected will be used exclusively for academic purposes, ensuring the anonymity of participants, in accordance with Resolutions 466/2012 and 510/2016 of the National Health Council.

Thank you for your attention, and I emphasize that this research has been approved by the Research Ethics Committee (CEP) of EERP-USP, which has the duty to ethically protect the research participant. At any stage of the study, you can request clarifications from the project coordinator, Prof. Dr. Fernanda Raphael Escobar Gimenes, by email at :

fregimenes@eerp.usp.br, or by phone at (16) 9 8140-5581. Further clarifications can be requested from the Research Ethics Committee of the School of Nursing of Ribeirão Preto at the University of São Paulo, by phone at (16) 3315-9197 or at Avenida dos Bandeirantes, 3900, University Campus - Monte Alegre Neighborhood, Ribeirão Preto - SP – Brazil, ZIP Code: 14040-902, from Monday to Friday – on business days – from 10:00 am to 12:00 pm and from 2:00 pm to 4:00 pm.

By agreeing to participate in the research, it is very important that you click on the link below and save a copy of this form with you. If you prefer, you can also send a message to the researcher requesting a copy by email. To download the ICF, click on this link: XXXXXXXXXX

After reading this form and understanding the conditions of the research:

☐ I agree to participate

☐ I do not agree to participate

UNIVERSIDADE DE SÃO PAULO
ESCOLA DE ENFERMAGEM DE RIBEIRÃO PRETO
Centro Colaborador da OPAS/OMS para o Desenvolvimento da Pesquisa em Enfermagem
Avenida Bandeirantes, 3900 - Ribeirão Preto - São Paulo - Brasil - CEP 14040-902
Fone: 55 16 3315.3382 - 55 16 3315.3381 - Fax: 55 16 3315.0518
www.eerp.usp.br - eerp@usp.br

Informed Consent Form (ICF) Evaluation by Experts of Educational Videos on the Use of Point-of-Care Ultrasound by Nurses

You are being invited to participate as an expert in stage 2 of the research titled "Point-of-Care Ultrasound for Nurses: Development and Validation of Digital Educational Technologies." This project is linked to the School of Nursing of Ribeirão Preto at the University of São Paulo (EERP-USP) and led by Prof. Dr. Fernanda Raphael Escobar Gimenes.

Stage 2 aims to develop and analyze the validity evidence of educational videos focused on the use of point-of-care ultrasound for nurses. This is a cross-sectional methodological study. The research seeks to contribute to nursing practice, specifically in the context of using PoCUS in clinical care. Our main objective is to strengthen and expand Patient Safety within the nursing scope. To achieve this goal, we invite you to participate as an evaluator in the video validation process. Your evaluation will focus on the validity of content evidence and the visual presentation of this technology through the Educational Health Technology Appearance Validation Instrument (IVATES), which should be completed and returned within 30 days of receipt. The estimated time for the evaluation is approximately 40 minutes, at a day and time of your convenience.

To ensure your complete immersion in the topic, we will send a comprehensive package by email. This will include a questionnaire for expert characterization, the scripts and storyboards of the educational videos, the validation instrument, the video script, the IVATES, a method for evaluating the appearance of educational health technology, and the bibliographic references that served as the basis for creating the scripts and storyboards on the Moodle platform. Your feedback will play an essential role, significantly contributing to the advancement of this study.

The risks of this research are minimal and are mainly related to expressing your opinion and the time spent responding to the instrument's evaluation questionnaire. If you feel uncomfortable expressing your opinion, the researcher will be available to address your discomfort. Additionally, there are inherent risks in the virtual environment, such as technological limitations. However, at the end of the research, the information will be downloaded to a password-protected device accessible only to the researchers. The data on the virtual platform will be deleted. In case of damages resulting from participation in the research, we guarantee the right to compensation according to the laws in force in the country (Item IV.3-h – CNS Resolution 466/2012).

Your participation is voluntary and will not bring immediate personal benefits, but it will contribute to the knowledge in the field of nursing and may positively impact future patient care involving PoCUS procedures.

It is important to note that if at any time you decide not to participate in the research, you can withdraw your consent without any personal prejudice. Additionally, you have the right to

request the complete deletion of your information from the study. Furthermore, there will be no costs or remuneration for participation.

The information collected will be used exclusively for academic purposes, ensuring the anonymity of participants, in accordance with Resolutions 466/2012 and 510/2016 of the National Health Council.

Thank you for your attention, and I emphasize that this research has been approved by the Research Ethics Committee (CEP) of EERP-USP, which has the duty to ethically protect the research participant. At any stage of the study, you can request clarifications from the project coordinator, Prof. Dr. Fernanda Raphael Escobar Gimenes, by email at: fregimenes@eerp.usp.br , or by phone at (16) 9 8140-5581. Further clarifications can be requested from the Research Ethics Committee of the School of Nursing of Ribeirão Preto at the University of São Paulo, by phone at (16) 3315-9197 or at Avenida dos Bandeirantes, 3900, University Campus - Monte Alegre Neighborhood, Ribeirão Preto - SP – Brazil, ZIP Code: 14040-902, from Monday to Friday – on business days – from 10:00 am to 12:00 pm and from 2:00 pm to 4:00 pm.

By agreeing to participate in the research, it is very important that you click on the link below and save a copy of this form with you. If you prefer, you can also send a message to the researcher requesting a copy by email. To download the ICF, click on this link: XXXXXXXXXX

After reading this form and understanding the conditions of the research:

☐ I agree to participate

☐ I do not agree to participate

Avenida Bandeirantes, 3900 - Ribeirão Preto - São Paulo - Brasil - CEP 14040-902
Fone: 55 16 3315.3382 - 55 16 3315.3381 - Fax: 55 16 3315.0518
www.eerp.usp.br - eerp@usp.br

Informed Consent Form (ICF) Evaluation by Experts of a Virtual Learning Environment on the Use of Point-of-Care Ultrasound by Nurses

You are being invited to participate as an expert in stage 2 of the research titled "Point-of-Care Ultrasound for Nurses: Development and Validation of Digital Educational Technologies." This project is linked to the School of Nursing of Ribeirão Preto at the University of São Paulo (EERP-USP) and led by Prof. Dr. Fernanda Raphael Escobar Gimenes.

Stage 3 aims to develop and analyze the content and face validity evidence of a virtual learning object focused on the use of point-of-care ultrasound by nurses. This is a cross-sectional methodological study. The research seeks to contribute to nursing practice, specifically in the context of using PoCUS in clinical care. Our main objective is to strengthen and expand Patient Safety within the nursing scope. To achieve this goal, we invite you to participate as an evaluator in the video validation process. Your evaluation will focus on the validity of content evidence and the visual presentation of this technology through the Educational Health Technology Appearance Validation Instrument (IVATES), which should be completed and returned within 30 days of receipt. The estimated time for the evaluation is approximately 40 minutes, at a day and time of your convenience.

The risks of this research are minimal and are mainly related to expressing your opinion and the time spent responding to the instrument's evaluation questionnaire. If you feel uncomfortable expressing your opinion, the researcher will be available to address your discomfort. Additionally, there are inherent risks in the virtual environment, such as technological limitations. However, at the end of the research, the information will be downloaded to a password-protected device accessible only to the researchers. The data on the virtual platform will be deleted. In case of damages resulting from participation in the research, we guarantee the right to compensation according to the laws in force in the country (Item IV.3-h – CNS Resolution 466/2012).

Your participation is voluntary and will not bring immediate personal benefits, but it will contribute to the knowledge in the field of nursing and may positively impact future patient care involving PoCUS procedures.

It is important to note that if at any time you decide not to participate in the research, you can withdraw your consent without any personal prejudice. Additionally, you have the right to request the complete deletion of your information from the study. Furthermore, there will be no costs or remuneration for participation, which is voluntary.

The information collected will be used exclusively for academic purposes, ensuring the anonymity of participants, in accordance with Resolutions 466/2012 and 510/2016 of the National Health Council.

Thank you for your attention, and I emphasize that this research has been approved by the Research Ethics Committee (CEP) of EERP-USP, which has the duty to ethically protect the research participant. At any stage of the study, you can request clarifications from the project coordinator, Prof. Dr. Fernanda Raphael Escobar Gimenes, by email at: fregimenes@eerp.usp.br, or by phone at (16) 9 8140-5581. Further clarifications can be requested from the Research Ethics Committee of the School of Nursing of Ribeirão Preto at the University of São Paulo, by phone at (16) 3315-9197 or at Avenida dos Bandeirantes, 3900, University Campus - Monte Alegre Neighborhood, Ribeirão Preto - SP – Brazil, ZIP Code: 14040-902, from Monday to Friday – on business days – from 10:00 am to 12:00 pm and from 2:00 pm to 4:00 pm.

By agreeing to participate in the research, it is very important that you click on the link below and save a copy of this form with you. If you prefer, you can also send a message to the researcher requesting a copy by email. To download the ICF, click on this link: XXXXXXXXXX

After reading this form and understanding the conditions of the research:

☐ I agree to participate

☐ I do not agree to participate
